# Experimental and numerical study on the transient temperature response and evolution during near-wall collapse of cavitation bubble

**DOI:** 10.1016/j.ultsonch.2026.108057

**Published:** 2026-09-17

**Authors:** Minglei Shan, Mengyu Feng, Ling Kuai, Yu Yang, Cheng Yin, Qingbang Han

**Affiliations:** a College of Information Science and Engineering, Hohai University, Changzhou, 213200, China; b College of Information Science and Technology & College of Artificial Intelligence, Nanjing Forestry University, Nanjing, 210037, China

**Keywords:** High-speed thin-film thermocouple, MRT-LBM, Transient temperature measurement, Cavitation bubble collapse

## Abstract

The near-wall collapse of cavitation bubbles is accompanied by high-speed micro-jets, localized high pressure, and transient thermal effects. The transient temperature evolution is difficult to measure directly because of its microsecond time scale and highly localized thermal response. Therefore, the temperature evolution and energy-transfer mechanism under near-wall conditions remain insufficiently understood. In this study, a combined experimental and numerical approach is adopted to investigate the transient temperature response during near-wall cavitation bubble collapse. A microsecond-resolved high-speed thin-film thermocouple system is developed for direct wall-temperature measurement during cavitation bubble collapse. A thermal-nucleation model based on the hybrid thermal multi-relaxation-time lattice Boltzmann method (MRT-LBM) is established to analyze and verify the corresponding thermal mechanism. The hybrid thermal MRT-LBM model is validated under free-field conditions by comparing the simulated bubble-radius evolution and temperature response with the experimental measurements. The asymmetric collapse, and wall-temperature evolution under near-wall conditions are then analyzed. The influence of the dimensionless stand-off distance γ on the thermal response is also examined. Experimental and numerical results indicate that the instantaneous temperature response during the near-wall collapse of cavitation bubbles can be generally divided into four stages: the shock wave-induced temperature rise phase, the liquid film compression-induced temperature rise phase, the cavitation cooling phase, and the post-cavitation plateau phase. We have analyzed the mechanisms underlying the temperature response in each stage, supported by experimental or numerical results. This study presents two valuable findings. For the first time, we measured temperature changes induced by a shock wave with a temperature change rate on the order of 10^6^ K/s and the cavitation cooling effect with a cooling rate on the order of 10^5^ K/s. These discoveries validate the feasibility of the direct temperature measurement experimental scheme for cavitation bubble collapse based on microsecond-level high-speed thermocouples, elucidate the thermal response mechanism of near-wall cavitation bubble collapse, and establish a theoretical and experimental foundation for studying thermal damage caused by cavitation bubble collapse.

## Highlights

• Microsecond thin-film thermocouple measures cavitation bubble temperature directly.
• We directly observed 10^6^ K/s shock heating and 10^5^ K/s cavitation cooling rates.
• Optimal wall heating occurs at stand-off distance 0.75 for near-wall bubble collapse.

## 1 Introduction

Cavitation bubble collapse involves extreme physical effects, including high-speed micro-jets, localized high pressure, and transient high temperature [1]. These effects are widely encountered in cavitation erosion, ultrasonic cleaning, heat-transfer enhancement, and biomedical processes [2], [3], [4]. Compared with the nearly spherically symmetric collapse in a free field, the presence of a solid boundary breaks the flow-field symmetry under near-wall conditions and promotes the formation of a high-speed micro-jet directed toward the wall during the late stage of collapse. This process not only substantially enhances the local impact pressure but also induces complex thermo-fluid coupling. Therefore, the thermal effect of near-wall cavitation has become an important issue in cavitation dynamics [5].

Current studies on near-wall cavitation bubble collapse have mainly focused on bubble morphology evolution, micro-jet formation, impact pressure, and cavitation erosion. For example, Zeng et al. showed that the stand-off distance significantly affects collapse asymmetry and micro-jet intensity, thereby modifying the distribution of impact pressure on the wall and the cavitation-erosion characteristics [5]. Scholars have extended the research focus on near-wall cavitation bubble collapse from rigid walls to elastic boundaries, revealing the complex effects of temperature, wall stiffness, and stand-off distance on jet morphology and wall stress [6], [7], [8]. In contrast, the transient thermal response and its formation mechanism during near-wall bubble collapse remain relatively less explored. In particular, a systematic understanding is still lacking of how local heat is generated, evolves, and dissipates after micro-jet impact on the wall, and of the physical mechanism responsible for the formation of a high-temperature plateau near the wall.

Experimental investigation of cavitation thermal effects is challenging. Cavitation bubbles are typically on the millimeter or even micrometer scale, whereas the collapse process lasts only from microseconds to milliseconds, making it difficult for conventional thermometry to directly capture the transient thermal response. As early as 1952, Chesterman proposed an experimental device in which a water-filled perspex tube was abruptly stopped to generate cavitation bubbles, enabling observation of cavitation induced by pressure variations in the liquid. This method was subsequently termed the “tube-arrest” or “Chesterman” method [9]. Dular combined the tube-arrest method with infrared thermography and observed a temperature decrease of approximately 3 K during bubble growth and an increase of approximately 4 K during collapse [10]. However, the inner diameter of the glass tube in the conventional tube-arrest apparatus is usually small, and the bubble growth is readily constrained by the tube wall. The resulting condition is therefore not an ideal unbounded liquid field. In addition, the spatiotemporal resolution and temperature sensitivity of infrared thermometry remain insufficient for resolving small transient temperature variations. Pulsed-discharge bubble generation produces vapor bubbles by applying a voltage pulse between underwater electrodes and using Joule heating to vaporize the surrounding liquid. This approach can generate relatively large cavitation bubbles on the millimeter or even centimeter scale, which facilitates optical observation. The discharge parameters, including voltage and current, are also adjustable, allowing partial control of the initial bubble energy. The experiments of Vokurka and Plocek confirmed the similarity in thermal behavior among spark-generated, ultrasonic, and laser-induced bubbles [11]. Zhang et al. generated cavitation bubbles using underwater pulsed discharge and estimated a flash temperature of approximately 7000 K from sonoluminescence spectra using a two-line method [12]. However, to the best of our knowledge, no published study has obtained the temperature response and evolution during near-wall cavitation bubble collapse through direct temperature measurement.

In addition to the difficulty of generating a controllable single cavitation bubble, capturing the transient temperature variation during its extremely short collapse process is another central experimental challenge. Existing temperature-measurement techniques can be broadly divided into indirect and direct methods. Indirect approaches, such as sonoluminescence spectroscopy [13], infer the internal temperature from the emission spectrum during bubble collapse. This approach relies on sufficient emission intensity and is experimentally complex, whereas chemical-probe methods [14] suffer from low spatial resolution and multiple interfering factors. Direct methods aim to obtain more direct signals through contact measurements using physical sensors, such as thermocouples [15], thermal resistors [16], and optical fibers [17]. However, the response time of conventional wire thermocouples is typically on the millisecond scale, which is too slow for the microsecond-scale transients of cavitation collapse. Their relatively large size also causes non-negligible disturbance to microscale flow fields. To overcome this limitation, high-speed thin-film thermocouple technology has emerged as a promising solution [18]. This technique uses micro- and nanofabrication processes to deposit thermocouple materials with micrometer- or nanometer-scale thickness on an insulating substrate, forming a thin-film sensing element with extremely low thermal capacity. Its response time can reach the microsecond or even nanosecond scale, while maintaining low spatial invasiveness. Researchers have successfully applied this technique to transient surface-temperature measurements in combustion [19], engine blades [20], and extreme machining [21]. However, applying high-speed thin-film thermocouples to cavitation bubble collapse presents unique challenges. The sensor must operate in a complex underwater flow field and withstand intense collapse-induced pressure impacts, as well as possible interference from phase transition. More importantly, when high-voltage discharge is used for bubble generation, the strong electromagnetic pulse can interfere with the weak thermoelectric signal. Therefore, thin-film thermocouples offer clear advantages for transient temperature measurement. Nevertheless, systematic studies that use them to obtain direct, reliable, and interference-resistant temperature signals during cavitation bubble collapse remain rare, especially under near-wall conditions.

Numerical simulation has meanwhile become an important tool for investigating cavitation thermal effects. Compared with conventional continuum methods, the lattice Boltzmann method (LBM) offers clear advantages in treating multiphase-interface evolution, complex boundaries, and multiphysics coupling. It has been widely used to study cavitation bubble growth and collapse [22], [23]. Scholars have used the thermal lattice Boltzmann method to investigate the nucleation, growth, interaction, and collapse dynamics of cavitation bubbles under thermodynamic coupling, with particular emphasis on the mechanisms of asymmetric jet formation, pressure-wave superposition, and phase-change thermal effects between bubbles. These studies provide a foundation for our investigation of the temperature response during cavitation bubble collapse near solid walls [24], [25].

Studies that combine high-speed transient thermometry with thermal LBM simulation to provide coupled experimental–numerical validation of near-wall cavitation thermal effects are still scarce [26]. Motivated by these issues, this study combines microsecond-resolved high-speed thin-film thermocouple experiments with hybrid thermal MRT-LBM simulations to investigate the transient thermal response during near-wall cavitation bubble collapse. First, the effectiveness of the experimental platform and numerical method is validated through the bubble dynamics and temperature evolution during single-bubble collapse under free-field conditions. Then, the inception, growth, and collapse of an asymmetric cavitation bubble near a solid wall, together with the resulting transient wall-temperature response, are investigated. Finally, a generalized pattern of the transient wall-temperature response is established, and the mechanism governing wall-temperature evolution is analyzed on the basis of the experimental and numerical results.

The remainder of this paper is organized as follows. Section 2 describes the experimental system, temperature-measurement method, and hybrid thermal MRT-LBM model. Sections 3.1, 3.2 validates the model under free-field conditions and analyzes near-wall collapse characteristics. Sections 3.3, 3.4 discusses the wall-temperature response and energy-partition mechanism. Section 4 summarizes the main conclusions.

## 2 Experimental system and numerical method

### 2.1 Experimental system

To investigate the transient thermal response during near-wall cavitation bubble collapse, an experimental platform was constructed. As shown in Fig. 1, the platform integrates controllable bubble generation, high-speed dynamic visualization, and microsecond-resolved temperature measurement. A transparent acrylic tank was used as the liquid container to enable visualization of bubble growth and collapse. Degassed deionized water was used as the working medium, and the ambient temperature was kept stable during the experiments to reduce the influence of environmental disturbances on the temperature measurements.

Cavitation bubbles were generated by low-voltage underwater pulsed discharge. The discharge system mainly consisted of adjustable DC power supply, an energy-storage capacitor, and discharge electrodes. A thin copper wire with a diameter of 0.2 mm and a local defect at the center was used as the discharge electrode. The energy-storage capacitance was 6800μF, and the charging voltage did not exceed 80 V. During the experiment, the capacitor instantaneously released electrical energy, causing rapid local vaporization of the liquid between the electrodes to form an initial vapor nucleus, which then expanded into a cavitation bubble. Compared with conventional high-voltage breakdown, low-voltage pulsed discharge enables stable generation of a single millimeter-scale cavitation bubble. It also reduces discharge-induced electromagnetic interference and facilitates the acquisition of weak transient thermal signals. By adjusting the input voltage and capacitance parameters, the initial bubble energy and maximum radius can be controlled, ensuring stable and repeatable experimental conditions.

The cavitation bubble dynamics were recorded using an AcutEye CP90-3-M-540 high-speed camera at a frame rate of 12 012 fps. The high-speed camera, discharge system, and temperature-acquisition system were synchronized by a unified trigger signal to ensure temporal correspondence between bubble morphology evolution and temperature response. After the experiments, image processing was used to obtain dynamic parameters, including the bubble-radius variation, maximum radius, collapse time, and micro-jet formation time.

**Fig. 1:**
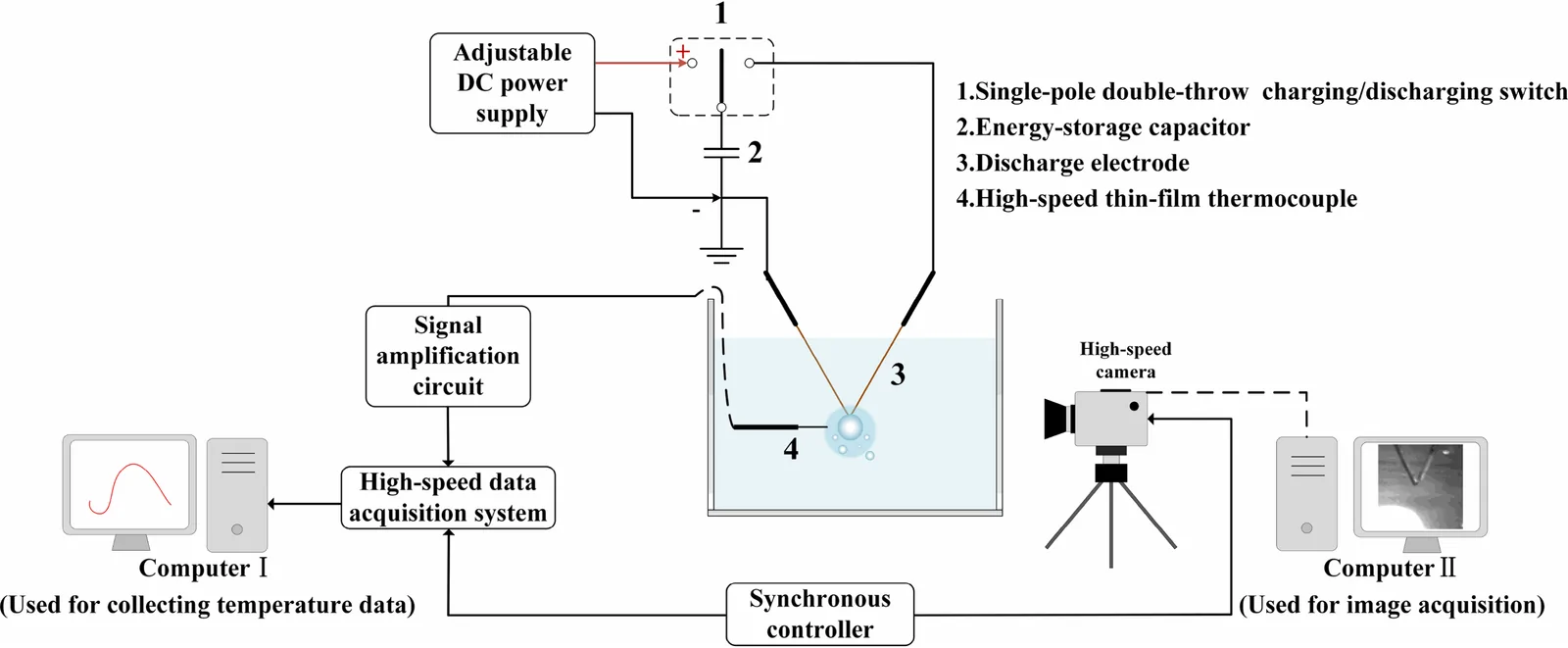
Schematic of the experimental platform.

Temperature was measured using custom needle-type high-speed thin-film thermocouples and bolt-armored needle-type high-speed thin-film thermocouple units fabricated in collaboration with Prof. Yunxian Cui’s group at Dalian Jiaotong University. The hot-junction diameter was 2 mm for both sensors. Pulse-laser calibration showed that the response time was less than 10μs, and the average error below 500 °C was within 0.5%, with a K-type thermocouple sensitivity of approximately 0.04 mV/°C, enabling rapid response to local liquid-temperature variations during cavitation bubble collapse. The thermocouple output was conditioned using an AD8495 low-noise amplifier module and synchronously acquired by a USB8536 high-speed data-acquisition card at a sampling rate of 10 MHz. To suppress electromagnetic interference generated by the discharge pulse, shielded cables were used, and the discharge device, signal-conditioning module, and data-acquisition system were grounded together. The circuit layout was also optimized to reduce transient interference coupling.

In the free-field experiments, the bubble-generation position was kept far from solid boundaries to minimize wall effects on bubble dynamics and to maintain an approximately spherically symmetric bubble during growth and collapse. The thermocouple was placed near the cavitation bubble to measure local temperature variations during free-field collapse. To avoid significant disturbance to bubble dynamics, the thermocouple probe size was much smaller than the maximum bubble scale, and direct contact between the bubble and the hot-junction region was avoided.

Compared with free-field conditions, near-wall cavitation bubble collapse undergoes pronounced asymmetric collapse under wall confinement and induces a high-speed micro-jet directed toward the wall. To measure the local thermal response near the wall, the high-speed thin-film thermocouple was fixed near the expected micro-jet impact region. Wall-contact measurements were used to directly record the local transient temperature peak and the subsequent evolution of the high-temperature plateau. By adjusting the distance between the initial bubble center and the wall, the thermal-response characteristics under different near-wall conditions were further investigated. This configuration enabled simultaneous acquisition of bubble-evolution data and the transient wall-temperature response, providing an experimental basis for the subsequent analysis of near-wall cavitation thermal effects.

### 2.2 Numerical method

The key to numerical simulation of cavitation is the accurate description of fluid motion, phase transition, and the coupling effect of the temperature field. To effectively simulate voltage-pulse-induced cavitation bubble generation, which involves transient phase transition, extreme physical fields, and complex interfaces, the LBM at the mesoscopic scale was adopted in this study. Unlike conventional computational fluid dynamics methods, LBM characterizes macroscopic flow by simulating the distribution and migration of fluid particle populations on discrete lattice nodes. It combines the physical intuitiveness of microscopic methods with the computational efficiency of macroscopic methods and is particularly suitable for simulating multiphase flows and complex boundaries. To improve computational robustness, the multiple-relaxation-time (MRT) model was used in this study. The core idea of the MRT model is to perform collision in moment space, where the distribution function is transformed into moments with physical meanings, such as density, momentum, and energy. Different relaxation times are assigned to different moments. In this way, the dissipation process of each physical quantity can be controlled more precisely, nonphysical oscillations can be effectively suppressed, and numerical stability can be significantly enhanced. The expression is given as follows [27]

(1) $$f_{\alpha }\left(x+e_{\alpha }\delta_{t},t+\delta_{t}\right)=f_{\alpha }\left(x,t\right)-{\left(M^{-1}\Lambda M\right)}_{\alpha \beta }\left(f_{\beta }-f_{\beta }^{eq}\right)$$

$$\;+\delta_{t}F_{\alpha }^{\prime }.$$

Here, M is an orthogonal transformation matrix. It is used to project the distribution function from velocity space to moment space. Λ is a diagonal matrix, and its diagonal elements are the relaxation factors corresponding to each moment. By independently adjusting these relaxation factors in moment space, the numerical stability of the model can be effectively enhanced. The last term $F_{\alpha }^{\prime }$ is the component of the force term $F^{\prime }$ in velocity space. In general, this force term consists of the fluid–fluid particle interaction force and the fluid–solid interaction force.

In the multiphase-flow simulation, the Shan–Chen pseudopotential model was adopted in this study to describe the interaction force between the gas and liquid phases [28]. This model is a mesoscopic force model. Its basic assumption is that a nonlocal interaction force depending on the local density exists between fluid particles. This force drives the system to undergo spontaneous phase separation and maintains a gas–liquid interface with a certain thickness. The standard expression for this interaction force $F_{\alpha }^{\prime }$ is given by [29]

(2) $$F_{\alpha }^{\prime }=-G\psi \left(x\right)\underset{\alpha }{\sum }\omega \left({\left|e_{\alpha }\right|}^{2}\right)\psi \left(x+e_{\alpha }\right)e_{\alpha },$$

G denotes the interaction strength. In the D2Q9 model, the weights $\omega \left({\left|e_{\alpha }\right|}^{2}\right)$ are specifically given as ω(0)=0, ω(1)=1/3, and ω(2)=1/12. The expression of the pseudopotential function ψ(x) is [29]

(3) $$\psi \left(x\right)=\sqrt{\frac{2\left(P_{\mathrm{EOS}}-\rho c_{s}^{2}\right)}{Gc^{2}}},$$

G can take the value of ±1, which ensures that the value under the square root is positive. c is the lattice speed, which is generally taken as c = 1. $c_{s}=\sqrt{c^{2}/3}$ is the lattice sound speed. $P_{\mathrm{EOS}}$ denotes the pressure, through which various equations of state can be introduced. To describe the thermodynamic behavior of real fluids more accurately, especially the gas–liquid phase-transition process at the high density ratio involved in this study, the commonly used van der Waals equation of state was not adopted. Instead, the Carnahan–Starling (C–S) equation of state was employed. By introducing a correction term based on the hard-sphere model, this equation of state can better reflect the repulsive effect of dense fluids, thereby obtaining saturation density ratios and interfacial tension closer to actual values [30]. Its expression is

(4) $$P_{\mathrm{EOS}}=\rho R_{g}T\frac{1+\frac{b\rho }{4}+{\left(\frac{b\rho }{4}\right)}^{2}-{\left(\frac{b\rho }{4}\right)}^{3}}{{\left(1-\frac{b\rho }{4}\right)}^{3}}-a\rho^{2}.$$

In this equation, T denotes temperature. The parameters $a=0.4963{\left(R_{g}T_{c}\right)}^{2}/P_{c}$ and $b=0.1873R_{g}T_{c}/P_{c}$ denote the attraction and repulsion parameters between fluid particles, respectively. $T_{c}$ and $P_{c}$ represent the critical temperature and critical pressure, respectively. In the model used in this study, the relevant parameters are set as $R_{g}=1$, a=1, and b=4 [30].

In the model construction, the D2Q9 model proposed by Qian et al. was used in this study. This model has two spatial dimensions and nine discrete velocity directions [31]. The velocity components in different directions are

(5) $$e_{\alpha }=\left\{\begin{array}{cc} \left(0,0\right)\; & \alpha =0,\\ \left(\cos\left[\left(\alpha -1\right)\frac{\pi }{2}\right],\sin\left[\left(\alpha -1\right)\frac{\pi }{2}\right]\right)\; & \alpha =1,2,3,4,\\ \sqrt{2}\left(\cos\left[\left(2\alpha -1\right)\frac{\pi }{4}\right],\sin\left[\left(2\alpha -1\right)\frac{\pi }{4}\right]\right)\; & \alpha =5,6,7,8. \end{array}\right)$$

The equilibrium distribution function $f_{\alpha }^{eq}$ of this model can be expressed as

(6) $$f_{\alpha }^{eq}=\omega_{\alpha }\rho \left(1+\frac{e_{\alpha }\cdot u}{c_{s}^{2}}+\frac{{\left(e_{\alpha }\cdot u\right)}^{2}}{2c_{s}^{4}}-\frac{u^{2}}{2c_{s}^{2}}\right).$$

$\omega_{\alpha }$ is the weight coefficient, which is specifically expressed as

(7) $$\omega_{\alpha }=\left\{\begin{array}{cc} \frac{4}{9}\; & \alpha =0,\\ \frac{1}{9}\; & \alpha =1,2,3,4,\\ \frac{1}{36}\; & \alpha =5,6,7,8. \end{array}\right)$$

The macroscopic density ρ and macroscopic velocity u of the model are defined as

(8) $$\rho =\underset{\alpha }{\sum }f_{\alpha },$$

(9) $$u=\frac{1}{\rho }\underset{\alpha }{\sum }f_{\alpha }e_{\alpha },$$

In the D2Q9 model, the orthogonal transformation matrix M is expressed as

(10) $$M=\left[\begin{array}{ccccccccc} 1 & 1 & 1 & 1 & 1 & 1 & 1 & 1 & 1\\ -4 & -1 & -1 & -1 & -1 & 2 & 2 & 2 & 2\\ 4 & -2 & -2 & -2 & -2 & 1 & 1 & 1 & 1\\ 0 & 1 & 0 & -1 & 0 & 1 & -1 & -1 & 1\\ 0 & -2 & 0 & 2 & 0 & 1 & -1 & -1 & 1\\ 0 & 0 & 1 & 0 & -1 & 1 & 1 & -1 & -1\\ 0 & 0 & -2 & 0 & 2 & 1 & 1 & -1 & -1\\ 0 & 1 & -1 & 1 & -1 & 0 & 0 & 0 & 0\\ 0 & 0 & 0 & 0 & 0 & 1 & -1 & 1 & -1 \end{array}\right],$$

The diagonal matrix Λ is expressed as

(11) $$\Lambda =\mathrm{diag}\left(\tau_{\rho }^{-1},\tau_{e}^{-1},\tau_{\zeta }^{-1},\tau_{j}^{-1},\tau_{q}^{-1},\tau_{j}^{-1},\tau_{q}^{-1},\tau_{\nu }^{-1},\tau_{\nu }^{-1}\right).$$

Here, $\tau_{\rho }^{-1}$, $\tau_{e}^{-1}$, $\tau_{\zeta }^{-1}$, $\tau_{j}^{-1}$, $\tau_{q}^{-1}$, and $\tau_{\nu }^{-1}$ denote the relaxation-time factors associated with density, energy, the square of energy, momentum-flux components, energy-flux components, and the pressure tensor, respectively. The evolution of fluid particles consists of collision and streaming processes, which can be specifically expressed as

(12) $$f_{\alpha }^{*}\left(x,t\right)=f_{\alpha }\left(x,t\right)-{\left(M^{-1}\Lambda M\right)}_{\alpha \beta }\left(f_{\beta }-f_{\beta }^{eq}\right)+\delta_{t}F_{\alpha }^{\prime },$$

(13) $$f_{\alpha }\left(x+e_{\alpha }\delta_{t},t+\delta_{t}\right)=f_{\alpha }^{*}\left(x,t\right).$$

Here, $f_{\alpha }^{*}\left(x,t\right)$ denotes the distribution function obtained after the collision step of the fluid particles. Through the predefined orthogonal transformation matrix M, the distribution function can be mapped from the discrete velocity space to moment space. The collision process can be expressed in moment space as

(14) $$m^{*}=m-\Lambda \left(m-m^{eq}\right)+\delta_{t}\left(I-\frac{\Lambda }{2}\right)S,$$

In this equation, m=Mf, $m^{eq}=Mf^{eq}$, I denotes the unit tensor, and S is the force term in moment space. For the D2Q9 model, $m^{eq}$ can be expressed as

(15) $$m^{eq}=\rho (1,\;-2+3{\left|u\right|}^{2},\;1-3{\left|u\right|}^{2},\;u_{x},\;-u_{x},$$

$${u_{y},\;-u_{y},\;u_{x}^{2}-u_{y}^{2},\;u_{x}u_{y})}^{T}.$$

$u_{x}$ and $u_{y}$ denote the components of the macroscopic velocity u in the two spatial directions, respectively. However, when the standard Shan–Chen model is combined with the C–S equation of state, thermodynamic inconsistency often appears at high density ratios. For example, the Maxwell equal-area rule is not strictly satisfied, causing the densities on both sides of the interface to deviate from the theoretical values. To solve the thermodynamic-consistency problem at high density ratios, the improved forcing scheme proposed by Li et al. was adopted in this study [32]. The core of this scheme is to introduce an adjustable parameter ɛ related to the interface-normal gradient into the force term S in moment space. Its expression is

(16) $$S=\left[\begin{array}{c} 0\\ 6\left(u_{x}F_{x}^{\prime }+u_{y}F_{y}^{\prime }\right)+\frac{0.75\;ɛ{\left|F\right|}^{2}}{\psi^{2}\delta_{t}\left(\tau_{e}-0.5\right)}\\ -6\left(u_{x}F_{x}^{\prime }+u_{y}F_{y}^{\prime }\right)-\frac{0.75\;ɛ{\left|F\right|}^{2}}{\psi^{2}\delta_{t}\left(\tau_{\zeta }-0.5\right)}\\ F_{x}^{\prime }\\ -F_{x}^{\prime }\\ F_{y}^{\prime }\\ -F_{y}^{\prime }\\ 2\left(u_{x}F_{x}^{\prime }-u_{y}F_{y}^{\prime }\right)\\ u_{x}F_{y}^{\prime }+u_{y}F_{x}^{\prime } \end{array}\right].$$

By adjusting the value of ɛ, the equilibrium condition of the model can be effectively corrected. This improvement ensures the accuracy of interfacial dynamic behavior and is crucial for accurately simulating the extreme pressure and temperature at the instant of cavitation bubble collapse [32].

Based on the MRT-LBM framework described above, a thermal-nucleation numerical model was constructed in this section to simulate the whole process of cavitation bubble inception, growth, and collapse. Its reliability was verified from multiple perspectives. By analyzing the physical fields during bubble growth and collapse, the physical laws underlying the evolution process were further understood. The essence of the low-voltage pulsed-discharge bubble-generation method used in this study is that Joule heat is generated when the current passes through the contact resistance produced by the defective thin copper-wire electrode, causing the local liquid temperature to rise sharply. This extreme state causes the nearby liquid to undergo violent vaporization within an extremely short time. The large amount of vapor generated by liquid vaporization causes the local volume to expand rapidly and push the surrounding liquid outward, thereby forming a cavitation bubble. To simulate this temperature-driven phase-transition process, the hybrid thermal LBM method was adopted in this study to model the cavitation effect caused by Joule heating. The mass, momentum, and pressure fields of the fluid were solved by the pseudopotential MRT-LBM described above. The evolution of the temperature field was obtained by solving a temperature equation based on the law of energy conservation, which was discretized and solved using the conventional finite difference method.

**Fig. 2:**
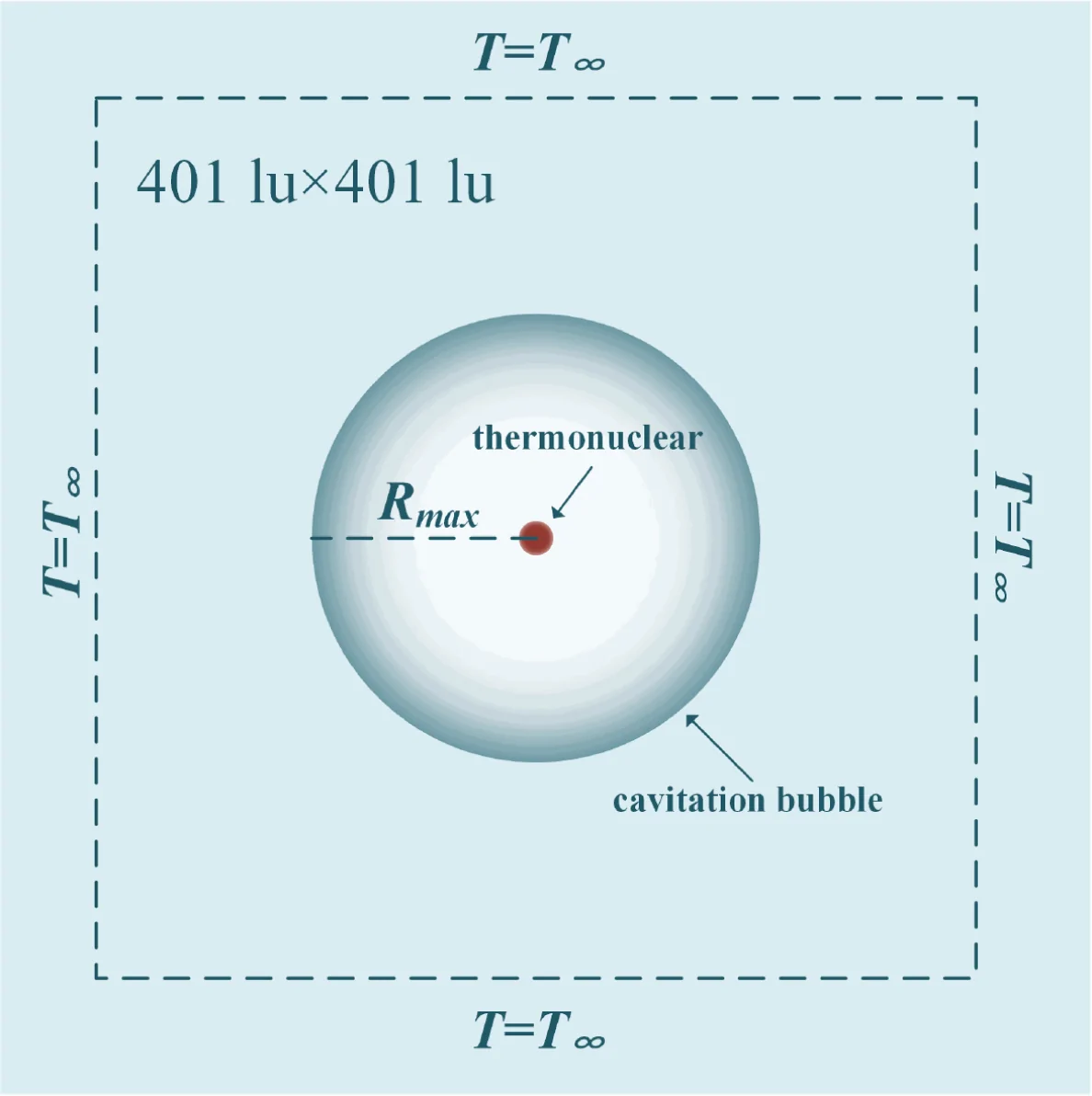
Schematic of the thermal-nucleation bubble-generation model.

The location of the thermal nucleus is the initialized high- temperature region that simulates energy injection. As shown in Fig. 2, a circular high-temperature region with a radius of $R_{tn}=10$ lattice units was set at the center of the computational domain of 401 × 401 lattice units. Its temperature $T_{tn}$ was much higher than the surrounding liquid temperature $T_{l}$. In addition, as described above, to simulate the simplest case, namely the evolution of a cavitation bubble in an unbounded liquid field, periodic boundary conditions were selected for the computational domain. The initialization formula of the temperature field is

(17) $$T\left(x,y\right)=\frac{T_{l}+T_{tn}}{2}+\frac{T_{l}-T_{tn}}{2}g\left(r\right),$$

the distribution function g(r) is used to describe the transition of temperature from the thermal-nucleus center to the surrounding liquid. The expression of r is

(18) $$r=\frac{2\left(\sqrt{{\left(x-x_{0}\right)}^{2}+{\left(y-y_{0}\right)}^{2}}-R_{tn}\right)}{W},$$

(x,y) denotes the position coordinates of the lattice nodes in the fluid domain, $\left(x_{0},y_{0}\right)$ denotes the position coordinates of the center of the thermal-nucleus model, and W denotes the interfacial thickness of the thermal nucleus. In this study, W was set to 5 lattice units. The thermal-nucleus distribution function was selected as an arctangent distribution, whose expression is

(19) $$g\left(r\right)=\frac{2}{\pi }\arctan\left(r\right).$$

To simulate the temperature-driven phase-transition process in which the high-temperature region induced by the thermal nucleus excites cavitation bubble formation, the temperature-field evolution in the flow domain must be solved. The pseudopotential MRT-LBM model used in this study can simulate the physical-field evolution process, including velocity, density, and pressure fields, only by solving the particle velocity distribution. The temperature equation was solved independently of the flow field by the finite difference method (FDM) and was coupled through the equation of state $P_{\mathrm{EOS}}$.

Starting from the local equilibrium law of entropy summarized by Anderson et al. and neglecting viscous dissipation, the equation can be expressed as [33]

(20) $$\rho T\frac{Ds}{Dt}=\nabla \cdot \left(\eta \nabla T\right).$$

s denotes entropy, and η is the thermal conductivity. Using the thermodynamic relation

(21) $$Tds=c_{v}dT+T{\left(\frac{\partial P_{\mathrm{EOS}}}{\partial T}\right)}_{\rho }d\left(\frac{1}{\rho }\right),$$

the temperature equation for solution can be derived. Here, $c_{v}$ is the specific heat capacity at constant volume. The temperature equation can be expressed as

(22) $$\rho c_{v}\frac{DT}{Dt}=\nabla \cdot \left(\eta \nabla T\right)-T{\left(\frac{\partial P_{\mathrm{EOS}}}{\partial T}\right)}_{\rho }\nabla \cdot u,$$

using ζ(T) to denote the right-hand side of the above equation gives

(23) $$\zeta \left(T\right)=-u\cdot \nabla T+\frac{1}{\rho c_{v}}\nabla \cdot \left(\eta \nabla T\right)-\frac{T}{\rho c_{v}}{\left(\frac{\partial P_{\mathrm{EOS}}}{\partial T}\right)}_{\rho }\nabla \cdot u,$$

the time discretization was performed using the fourth-order Runge–Kutta scheme

(24) $$T^{t+\delta t}=T^{t}+\frac{\delta_{t}}{6}\left(h_{1}+h_{2}+h_{3}+h_{4}\right),$$

$h_{1}$, $h_{2}$, $h_{3}$, and $h_{4}$ represent the slope estimates at different intermediate steps, respectively, and are expressed as

(25) $$h_{1}=\zeta \left(T^{t}\right),$$

$$h_{2}=\zeta \left(T^{t}+\frac{\delta_{t}}{2}h_{1}\right),$$

$$h_{3}=\zeta \left(T^{t}+\frac{\delta_{t}}{2}h_{2}\right),$$

$$h_{4}=\zeta \left(T^{t}+\delta_{t}h_{3}\right).$$

Therefore, at each time step, the density ρ and velocity u obtained from the pseudopotential MRT-LBM solution were substituted into the temperature equation to update the temperature field T. The new temperature field then influenced the local pressure through the equation of state, thereby driving phase transition and flow-field variation. Finally, dynamic coupling between temperature and phase transition was achieved.

## 3 Results and discussion

### 3.1 Model validation and thermal-response characteristics under free-field conditions

To validate the thermal-nucleation hybrid thermal MRT-LBM model, the free-field growth and collapse of a single cavitation bubble were first examined. Experimental and numerical comparisons were conducted to assess the ability of the model to predict cavitation bubble dynamics and transient thermal response. Both the experiment and simulation were configured under the same physical conditions to reproduce the generation, expansion, and collapse of a single cavitation bubble induced by local instantaneous energy deposition in a quiescent liquid.

Fig. 3 shows the experimental and numerical comparison of free-field bubble morphology evolution at a charging voltage of 60 V. The experimentally measured maximum bubble radius was approximately 5.38 mm, and the thermocouple measurement point was approximately 2.64 mm from the initial bubble center. Fig. 3(a) shows the bubble-evolution sequence captured by high-speed photography, and Fig. 3(b) shows the corresponding density-field distributions obtained from numerical simulation, where the white region represents the gas phase and the blue region represents the liquid phase.

The experimental and numerical results agree well during bubble growth, maximum expansion, and collapse. In the initial stage, the bubble expands approximately spherically. After reaching its maximum volume, it undergoes an axisymmetric collapse, and the bubble boundary rapidly contracts toward the center during the late stage. These results indicate that the established model can capture the main dynamic behavior of a free-field cavitation bubble.

**Fig. 3:**
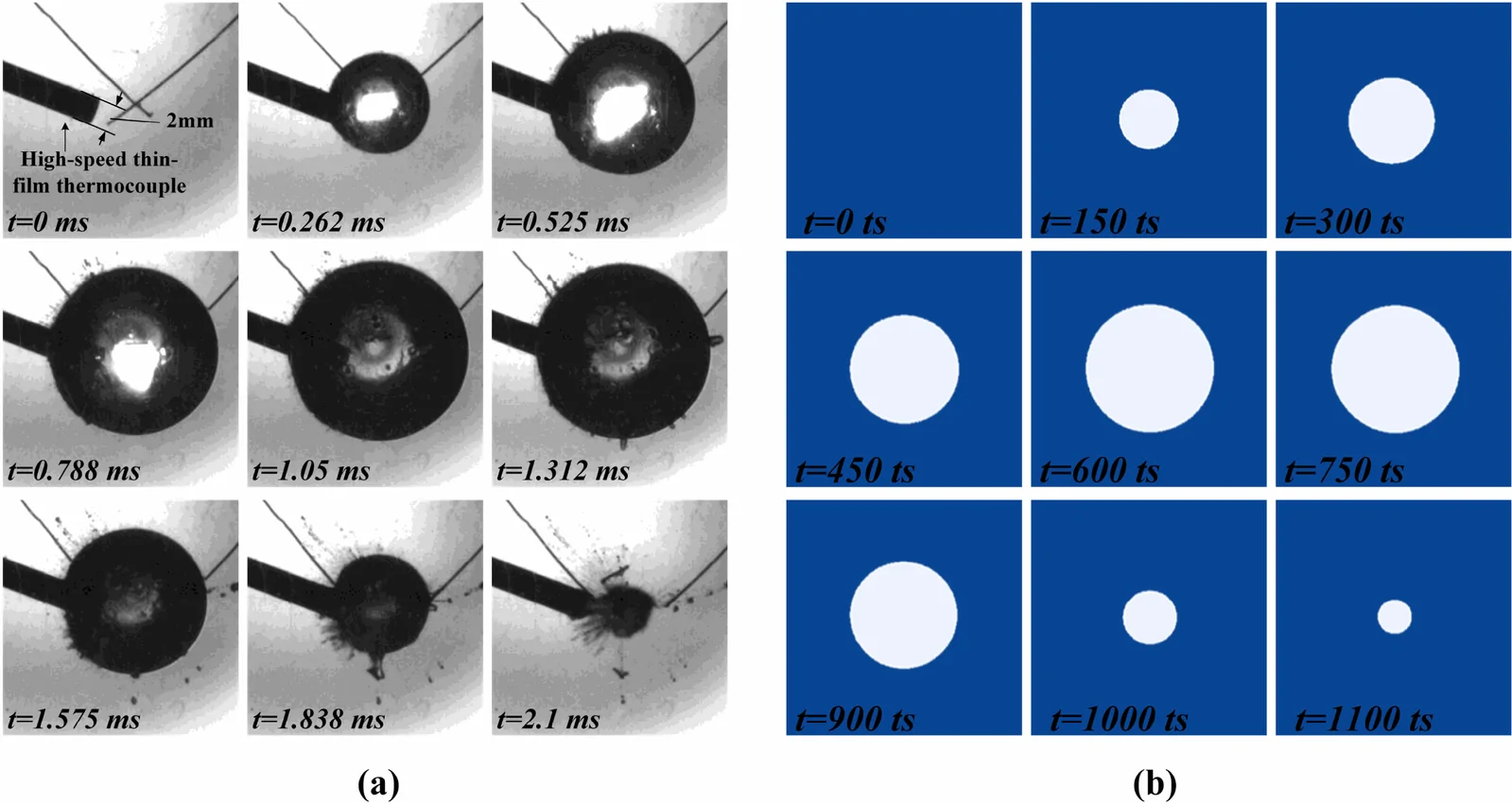
Temperature measurement of cavitation bubble collapse in an unbounded liquid field: (a) experimental images and (b) numerical simulation results.

Fig. 4 shows the experimental bubble-radius evolution under different operating conditions. The charging voltages were set to 40, 60, and 80 V. The maximum bubble radius increases noticeably with increasing charging voltage, rising from approximately 0.38 to 0.65 cm as the charging voltage increases from 40 to 80 V. Under all conditions, the bubble exhibits a typical rapid-expansion and slow-contraction dynamic behavior, and the overall shapes of the radius-evolution curves remain similar. With increasing charging voltage, the bubble reaches a larger maximum radius, while the radius evolves more gradually during the expansion process. This indicates that the input energy primarily affects the bubble size and the radius-evolution characteristics, without altering the fundamental dynamic evolution mechanism.

**Fig. 4:**
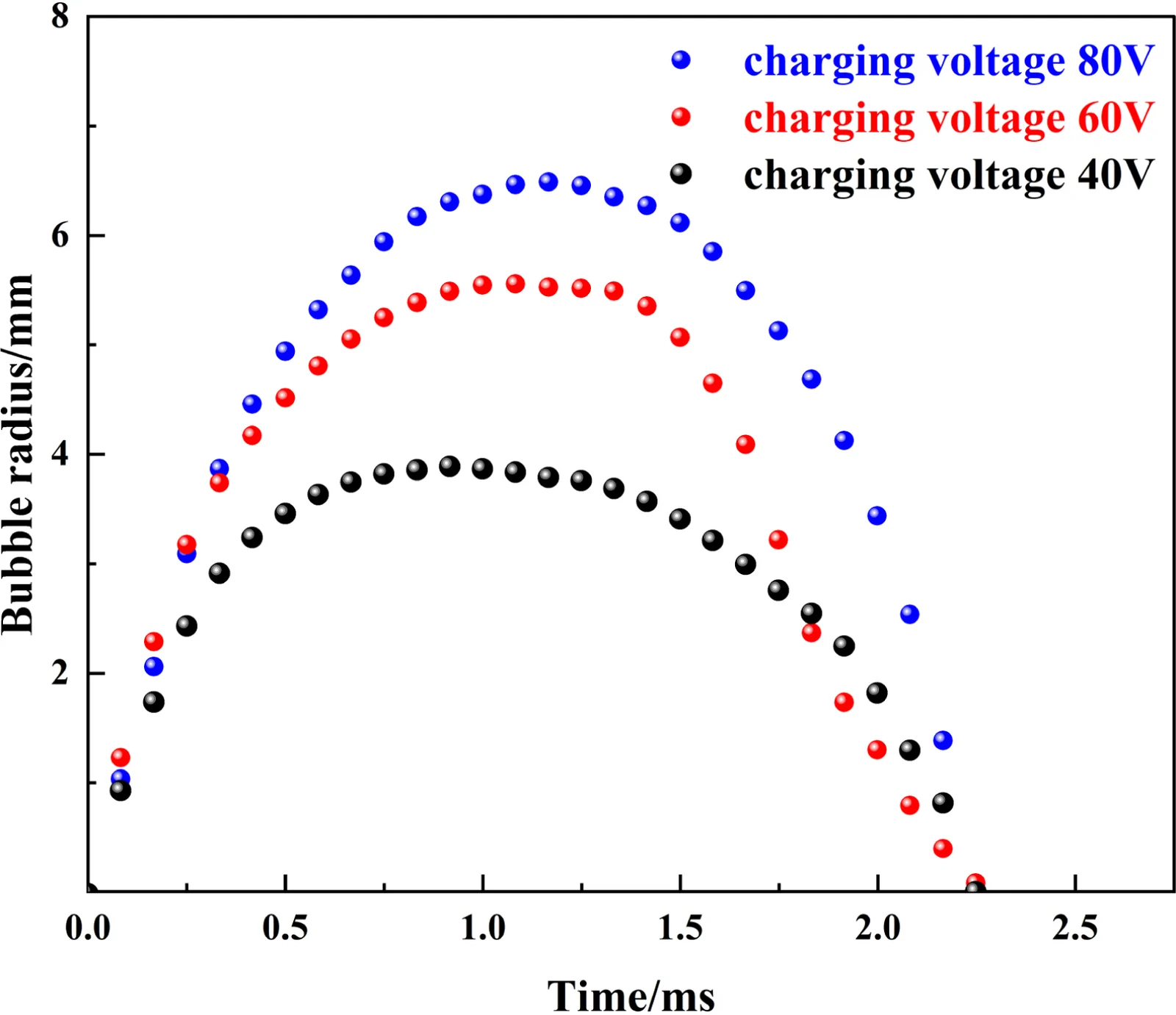
Bubble-radius evolution under different operating conditions.

After the dynamic validation, the transient thermal response during bubble collapse was further analyzed. Fig. 5 compares the experimentally measured temperature evolution with the numerical result for the same experiment presented in Fig. 3 at a charging voltage of 60 V. Therefore, the temperature signal can be directly correlated with the synchronized high-speed images shown in Fig. 3. The experimental signal was appropriately denoised, and the ratio between the measurement-point distance and the bubble-center distance in the numerical model was kept consistent with that in the experiment. The experimental and numerical results exhibit good agreement in the overall temperature-evolution trend, as shown in Fig. 3. This agreement further validates the ability of the proposed model to capture the transient thermal response associated with cavitation bubble collapse.

Both the experimental and numerical results exhibit a sharp temperature peak during the initial stage, characterized by a rapid temperature rise followed by a rapid decrease, as shown in Fig. 5. The entire temperature peak lasts for only approximately 240μs. In the experiment, its rising edge lasts approximately 50μs, and the measured temperature increase of approximately 52 °C corresponds to a temperature-rise rate on the order of 10^6^ K/s. Comparative measurements were conducted with the sensor immersed in water and positioned outside the water. The sharp temperature-rise signal was detected only when the sensor was immersed in water, thereby excluding electromagnetic interference and thermal radiation as possible causes of this signal. Because the duration of the temperature peak is comparable to that of a single crest of an underwater shock wave, both being on the order of 100μs, we further investigated whether this peak was induced by a shock wave. The hypothesis was examined through two independent approaches. First, the propagation speed was calculated from the distance between the bubble-generation hot spot and the sensor and the time required for the temperature peak to reach the sensor. Second, numerical simulations were performed to examine the temperature rise induced by shock-wave propagation through the liquid. The combined results show that the shock wave produces a pronounced temperature rise in the water. As shown in Fig. 6, Fig. 9, the propagation of the shock wave is accompanied by the synchronous propagation of a temperature peak. After rigorous temporal alignment, the propagation speed calculated from the experimental data, with measurement uncertainty taken into account, was found to be of the same order of magnitude as the speed of sound in water. Taken together, these results verify that the sharp temperature peak is induced by the shock wave.

To further elucidate the mechanism of transient heating during free-field bubble collapse, the evolution of the pressure, temperature, and velocity fields obtained from the numerical simulation is presented in Fig. 6. During the initial stage of cavitation inception, the high-temperature energy deposition induces abrupt changes in the density and pressure of the nearly incompressible liquid, generating a shock wave that propagates outward. The high pressure at the shock front strongly and approximately adiabatically compresses the liquid, thereby producing a pronounced transient temperature rise as the shock front passes through the medium. This process constitutes the underlying mechanism responsible for the sharp initial temperature peak observed in the experiment. It should be noted that the initial temperature peak predicted by the numerical simulation is less sharp than that measured experimentally. The experiment was conducted in real water, which is strongly resistant to compression, whereas the LBM model adopted in the numerical simulation is weakly compressible. Consequently, the intense adiabatic-compression-induced temperature response associated with shock-wave propagation is less pronounced in the numerical result.

**Fig. 5:**
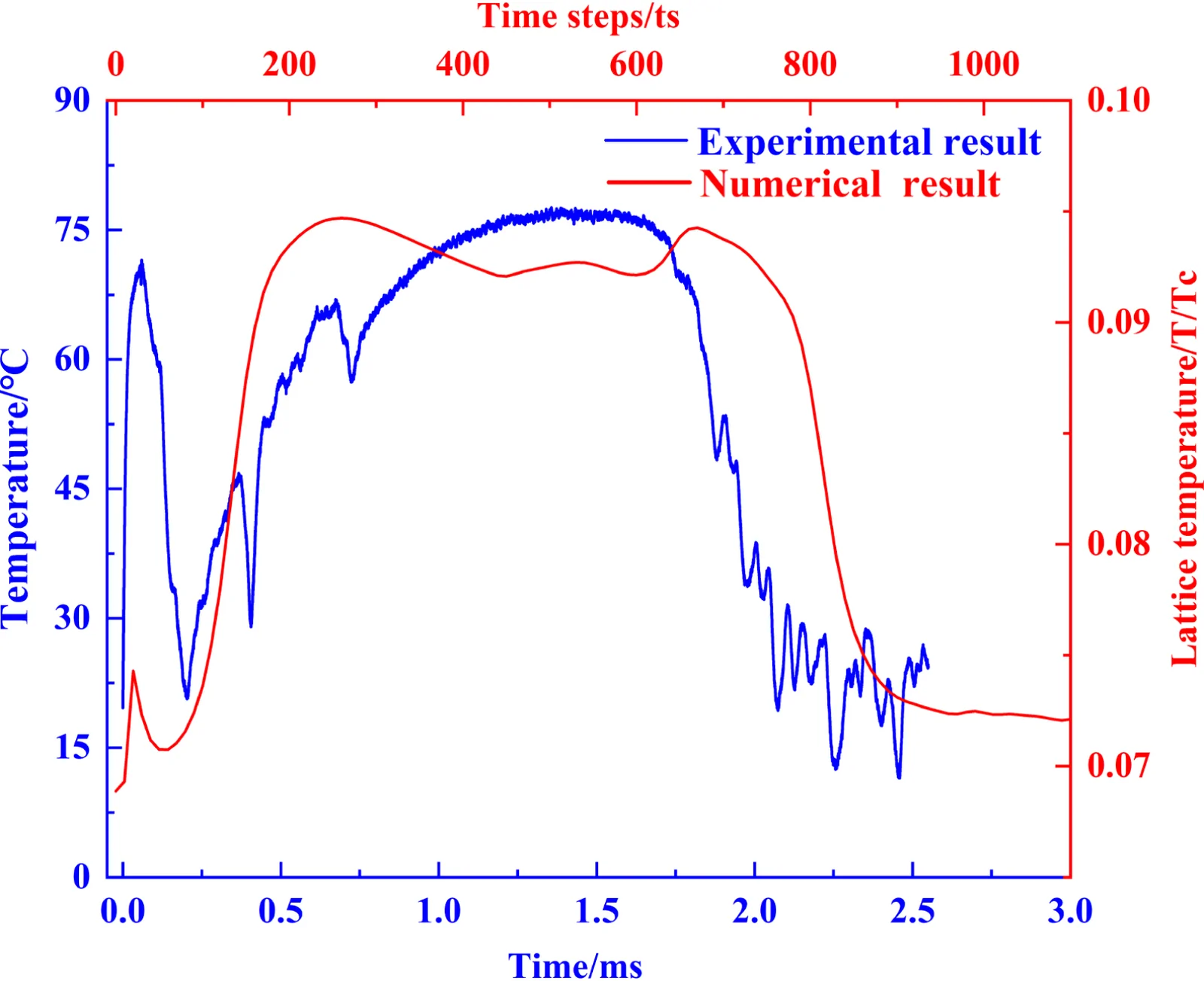
Comparison of temperature evolution during cavitation bubble collapse in an unbounded liquid field.

Further analysis shows that, under free-field conditions, the high-temperature region is mainly concentrated near the bubble center. Its formation mechanism primarily originates from adiabatic compression of intrabubble vapor during collapse. As the bubble contracts rapidly, the intrabubble pressure rises sharply, causing a synchronous increase in local temperature and forming a transient high-temperature thermal nucleus. After collapse, local heat is rapidly transferred to the surrounding liquid through thermal diffusion; therefore, the temperature pulse lasts only for a short duration.

**Fig. 6:**
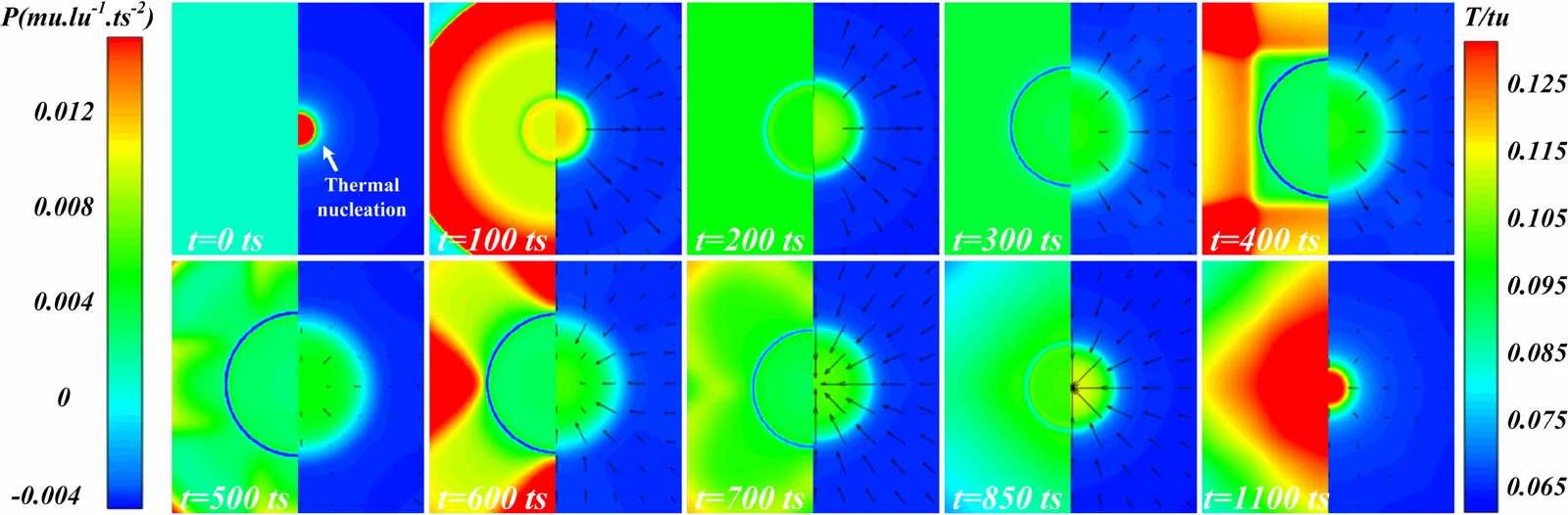
Numerical visualization of pressure, temperature, and velocity fields during free-field bubble collapse.

The experimental temperature signal exhibits certain high- frequency oscillations near the peak. These oscillations may originate from the transient impact of the collapse-induced shock wave on the thermocouple structure and from residual electromagnetic interference caused by the discharge pulse. By contrast, the numerical result is smoother and mainly reflects the intrinsic evolution of the coupled flow-thermal process. Although local differences exist between the two results, they agree well in key characteristics, including the temperature peak time, variation trend, and peak magnitude.

Overall, the bubble-dynamics and transient thermal-response results demonstrate that the established thermal-nucleation hybrid thermal MRT-LBM model can reproduce single-bubble generation, expansion, and collapse under free-field conditions. The model can also effectively capture the transient temperature rise during the collapse stage. These results validate the reliability of the model for describing free-field cavitation thermodynamics and provide a basis for the subsequent analysis of near-wall cavitation thermal effects.

### 3.2 Typical thermodynamic behavior of near-wall cavitation bubble collapse

Under free-field conditions, cavitation bubble collapse maintains strong spherical symmetry, and the thermal response mainly originates from intrabubble gas compression. Near-wall collapse of cavitation bubbles is a more commonly encountered phenomenon. The interaction between a collapsing cavitation bubble and a solid boundary provides a fundamental hydrodynamic and thermodynamic basis for applications such as cavitation cleaning and ultrasound-based medical treatments.

To investigate the thermodynamic behavior of bubble collapse under near-wall conditions, experimental and numerical studies were conducted on the basis of the free-field validation. The dimensionless stand-off distance was used to describe the relative position between the bubble and the wall. It is defined as $\gamma =s/R_{\max}$, where s is the vertical distance from the initial bubble center to the wall and $R_{\max}$ is the maximum bubble radius measured under the same free-field condition. Bubbles were still generated by low-voltage underwater discharge. To measure the transient wall temperature, a wall-integrated temperature-measurement unit was designed. The high-speed thin-film thermocouple was embedded inside a stainless-steel bolt, with the hot-junction plane flush with the front surface of the bolt. A thermally conductive insulating adhesive was used to fill the gap to ensure mechanical fixation and good thermal contact. The end face of the threaded end of the bolt served as the wall in the experiment. The outer diameter of the bolt was 10 mm, which is larger than the 2 mm hot-junction diameter. Therefore, it can provide a local rigid boundary and can also be regarded as a point-type wall-temperature measurement unit for direct measurement of the local wall thermal response. The working medium was deionized water, and the ambient temperature was maintained at 20±1°C.

A typical near-wall condition was first selected for analysis. The initial distance from the bubble center to the wall was approximately 6.3 mm, and the maximum bubble radius was approximately 12.5 mm, corresponding to γ≈0.50. Fig. 7 compares the experimental high-speed images and numerical density fields during near-wall bubble collapse. The collapse no longer maintains a spherically symmetric structure. The bubble initially expands in an approximately spherical manner, but as the bubble grows, the expansion on the wall side is suppressed, and the bubble gradually becomes asymmetric. During collapse, a nonzero Kelvin impulse is generated owing to the asymmetric constraint imposed by the rigid wall. The liquid on the side away from the wall has a higher contraction velocity, whereas liquid motion on the wall side is obstructed by the wall. Consequently, when the Kelvin impulse satisfies the Blake criterion for jet formation, the top of the bubble gradually indents inward, forming a high-speed liquid micro-jet directed toward the wall. The micro-jet subsequently penetrates the bubble and impacts the wall, and the collapse center shifts noticeably toward the wall.

**Fig. 7:**
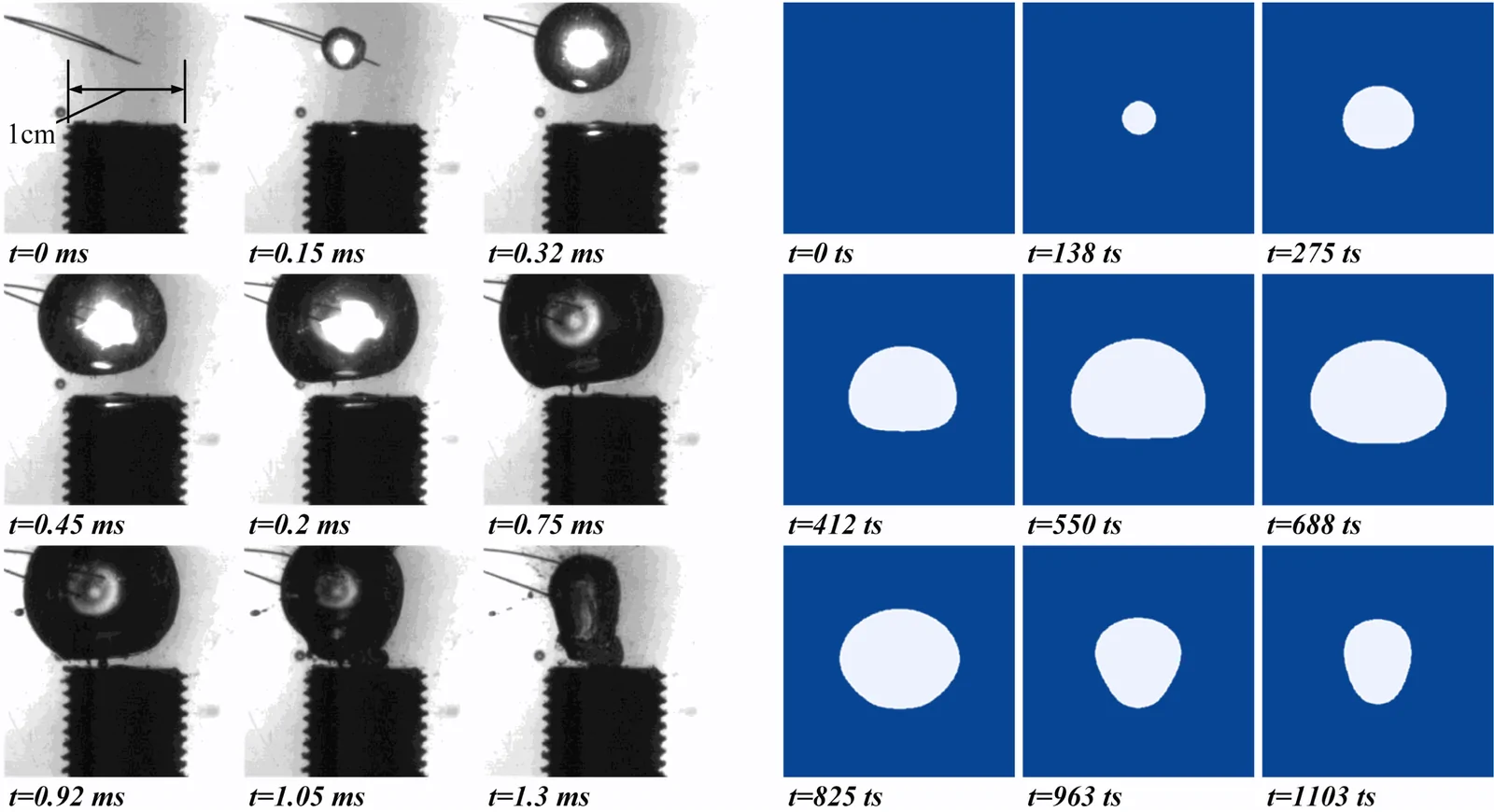
Temperature measurement of near-wall cavitation bubble collapse: (a) experimental images and (b) numerical simulation results.

The experimental and numerical results agree well in the overall deformation trend, micro-jet formation time, and jet direction, indicating that the established model can effectively describe asymmetric cavitation bubble collapse under near-wall conditions. Compared with the free-field case, the most prominent characteristic of near-wall cavitation is its wall thermal response. Fig. 8 shows the temperature evolution at the wall measurement point obtained experimentally and numerically. The wall-temperature curve under near-wall conditions exhibits a distinct four-stage thermal-response behavior.

The first-stage temperature rise occurs during the early stage of bubble generation. As in the free-field case, both the experimental and numerical results show a short transient temperature peak (peak 1) near the wall measurement point immediately after discharge. The profile and formation mechanism of peak 1 during near-wall cavitation bubble collapse are consistent with those observed under free-field conditions. Combined with the temperature- and velocity-field evolution, this temperature rise mainly originates from localized Joule heating generated when the discharge current passes through the high-resistance region of the thin copper wire immediately after the discharge pulse. The rapid thermal deposition heats the adjacent liquid within a very short time, induces local vaporization, and produces the first transient temperature peak near the wall measurement point. When the shock wave reaches the wall region, the local liquid undergoes transient compression, resulting in a rapid temperature rise followed immediately by a rapid decrease after the shock wave has passed. The second stage is characterized by a gradual temperature increase, as indicated by the experimental data between peak 1 and peak 2 in Fig. 8. This stage lasts for approximately 0.8 ms, during which the measured temperature increases by 16.7 °C, from 58.3 to 75.0 °C. It corresponds to the bubble-growth stage from t=0 ms to t=0.92 ms, as shown in Fig. 7. The third stage is characterized by a rapid temperature decrease. Beginning at peak 2, this stage lasts for approximately 0.5 ms, during which the temperature decreases rapidly from 75.0 to 25.0 °C. This stage corresponds to the collapse of the cavitation bubble. The fourth stage is a temperature plateau with only minor temperature variations, corresponding to the post-collapse stage of the cavitation bubble. The formation mechanism and evolution of the first stage have already been discussed in detail in Section 3.1, whereas the temperature variation during the fourth stage is relatively small and is not the primary focus of the present analysis. Therefore, the following analysis of the wall-temperature-response mechanism focuses mainly on the second and third stages. After the cavitation bubble begins its asymmetric near-wall collapse, as shown at t=1.05 ms in Fig. 7, the temperature measured at the measurement point exhibits several abrupt decreases.

**Fig. 8:**
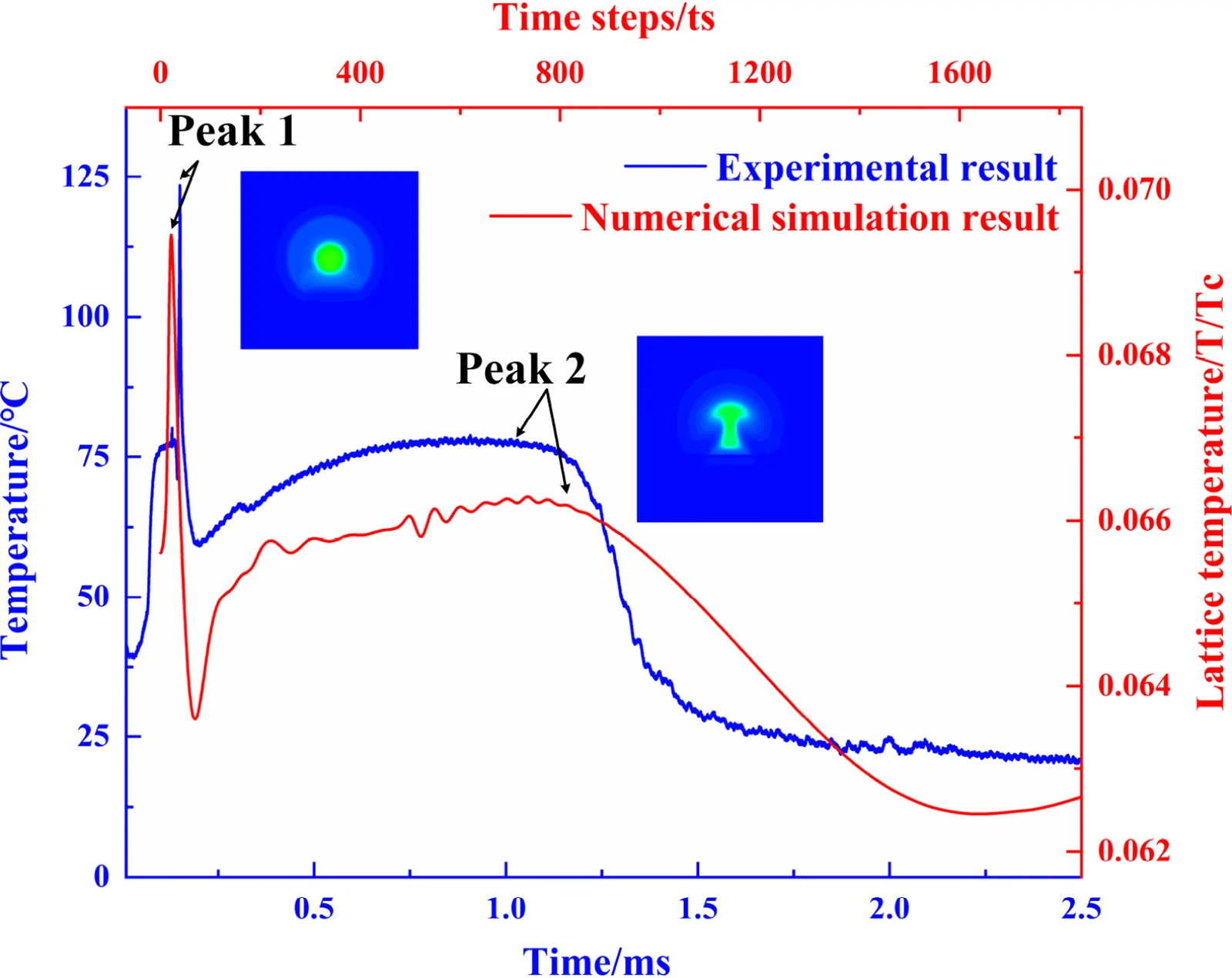
Comparison of temperature evolution during near-wall cavitation bubble collapse.

### 3.3 Mechanism of the thermal response during near-wall collapse

To clarify the temperature-response mechanism during near-wall cavitation bubble collapse, numerical simulations of the growth and collapse of a cavitation bubble at γ=0.5 were also conducted, as shown in Fig. 9. The temperature-response mechanism can be analyzed more clearly through a joint examination of Figs. 7, 8, and 9.

As discussed in Section 3.1, the sharp temperature peak in the first stage is caused by the shock wave. In this respect, there is no difference between the near-wall case and the free-field case; therefore, this mechanism is not discussed repeatedly here.

**Fig. 9:**
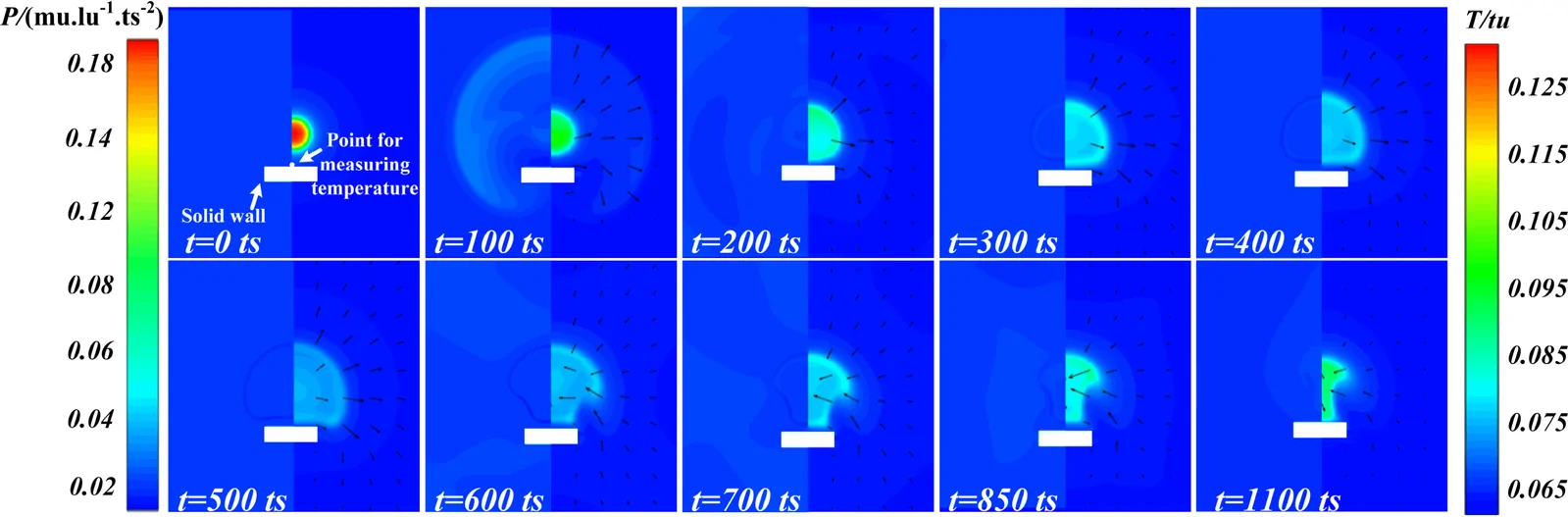
Numerical simulation of temperature-field evolution during near-wall cavitation bubble collapse at γ=0.5.

The second stage corresponds to the growth stage of the cavitation bubble. Inspired by the work of Ohl et al. [8], the temperature-measurement end face can be regarded as a no-slip boundary, and it is also treated in this manner in the numerical model. Owing to the no-slip boundary, a weakly flowing liquid film exists between the temperature-measurement end face and the cavitation bubble. The existence of this liquid film is confirmed by both the experimental and numerical results, as shown in Fig. 7, Fig. 9. During bubble growth, the bubble wall continuously compresses the liquid film against the temperature-measurement end face. Because the liquid film is difficult to remove completely, the temperature measured at the measurement point represents the temperature of the liquid film rather than that of the gas inside the cavitation bubble. As shown in Fig. 7, the cavitation bubble takes only approximately 0.75 ms to grow from its generation to its maximum size, and the compression velocity of the bubble wall is on the order of 10 m/s. For the liquid film between the bubble wall and the temperature-measurement end face, this process can be regarded as an approximately adiabatic compression process. During this process, the kinetic energy associated with the bubble motion is converted into the internal energy of the compressed liquid film, causing the liquid-film temperature to increase gradually while the bubble-wall velocity gradually decreases. When the cavitation bubble reaches its maximum size at approximately t=0.75 ms in Fig. 7, the bubble-wall velocity decreases to zero, and the measured temperature reaches its maximum value and remains at a plateau for a period.

The third stage corresponds to the collapse stage of the cavitation bubble. During collapse, the bubble wall contracts toward the interior of the bubble, placing the liquid film between the bubble wall and the temperature-measurement end face under bulk tension and producing a low-pressure region within the liquid film, as shown at $t=600t_{s}$ in Fig. 9. Because liquids have limited tensile strength and the temperature-measurement surface is not an ideally smooth plane owing to the sensor-installation requirements, nucleation and cavitation occur more readily at the measurement point, as shown at t=1.05 ms in Fig. 7. Cavitation is essentially a liquid-vaporization process that requires the absorption of a large amount of latent heat. It therefore produces severe local cooling at the temperature-measurement point, as shown by the experimental temperature curve in Fig. 8. This phenomenon is referred to as cavitation cooling.

The cooling process occurs rapidly, beginning with the collapse of the cavitation bubble at t=0.92 ms in Fig. 7, followed by the occurrence of cavitation at the temperature-measurement end face at t=1.05 ms and the subsequent intensification of cavitation at t=1.30 ms. The entire process lasts for approximately 0.38 ms, during which the temperature at the measurement point decreases from 76 °C at peak 2 to 25 °C, as shown by the experimental temperature curve in Fig. 8. The average cooling rate reaches approximately $1.3\times 10^{5}$ K/s, while the maximum slope of the cooling curve reaches approximately $2.5\times 10^{5}$ K/s. To the best of our knowledge, this is the first direct measurement of the cavitation-cooling rate.

The above analysis is based on the information presented in Figs. 7, 8, and 9 and considers only the case of γ=0.5. The dimensionless stand-off distance γ plays a crucial role in near-wall cavitation bubble collapse, and its influence on the corresponding temperature response requires further investigation.

### 3.4 Influence of stand-off distance on temperature-field evolution

In order to comprehensively investigate the temperature-response law of near-wall cavitation bubble collapse, a series of experiments was conducted with γ as the control variable, and 32 sets of experimental response curves were measured over the range 0.35≤γ≤1.85. Although measurements were not repeated multiple times at the same γ, the scatter statistics obtained at multiple different γ values can also demonstrate the non-randomness and scientific validity of the measurement data.

Fig. 10 shows 10 sets of temperature-measurement curves. It can be seen from these curves that all of them conform to the four stages defined above, indicating that they follow the same pattern and further confirming that the experimental data obtained in this study are not accidental.

As shown in Fig. 10(a), peak 2 increases with increasing γ. Peak 2 corresponds to the final stage of the second stage, namely, the final stage in which the cavitation bubble wall compresses the liquid film between the bubble wall and the temperature-measurement end face. When γ is small, such as when γ=0.5, the initial distance between the bubble wall and the temperature-measurement end face is relatively small, and compression of the liquid film therefore begins earlier. At this time, the temperature rise caused by the shock wave has not completely passed, so the starting temperature of the second stage is relatively high when γ is small. The slow temperature rise during the later part of this stage is similar to that observed under the other γ conditions. However, because a smaller γ corresponds to a shorter compression distance, the liquid film obtains less internal energy, resulting in a smaller temperature rise. As γ increases, the temperature rise obtained by the liquid film also gradually increases.

**Fig. 10:**
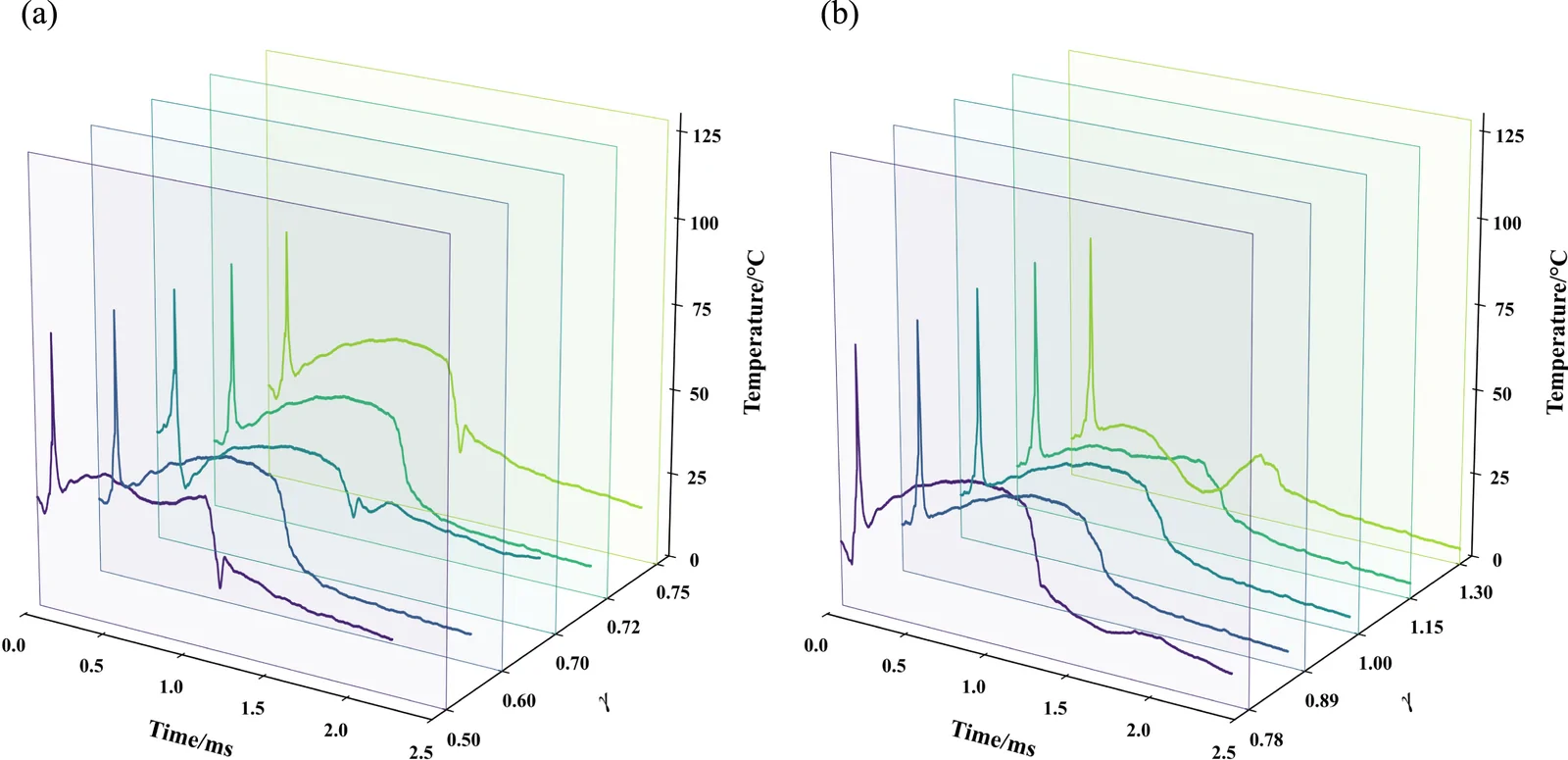
Experimental wall-temperature histories during near-wall cavitation bubble collapse at different dimensionless stand-off distances: (a) γ=0.50, 0.60, 0.70, 0.72, and 0.75; (b) γ=0.78, 0.89, 1.00, 1.15, and 1.30.

When γ≈0.75, compared with smaller γ, there is a longer compressible distance and a longer adiabatic compression time between the bubble wall and the temperature-measurement end face during the growth stage of the cavitation bubble. Therefore, under the same bubble-wall motion mode, more kinetic energy is converted into the internal energy of the liquid film, resulting in a higher temperature response.

When γ>0.75, a larger γ is accompanied by a thicker liquid film, which provides better fluidity, weakens the blocking effect of the no-slip temperature-measurement wall surface, and thus weakens the adiabatic compression process of the liquid film, resulting in a smaller temperature rise.

As shown in Fig. 11, in the low-γ region, the value of peak 2 increases with increasing γ, reaches its maximum value at γ≈0.75, and then decreases with further increasing γ. It is worth noting that the magnitude of peak 1 remains relatively stable. Because peak 1 is caused by shock waves, the temperature rise caused by shock waves is directly related to the shock-wave energy. Therefore, the basic invariance of peak 1 in Fig. 11 indirectly verifies that the discharge energy of each discharge-induced bubble was approximately equivalent.

**Fig. 11:**
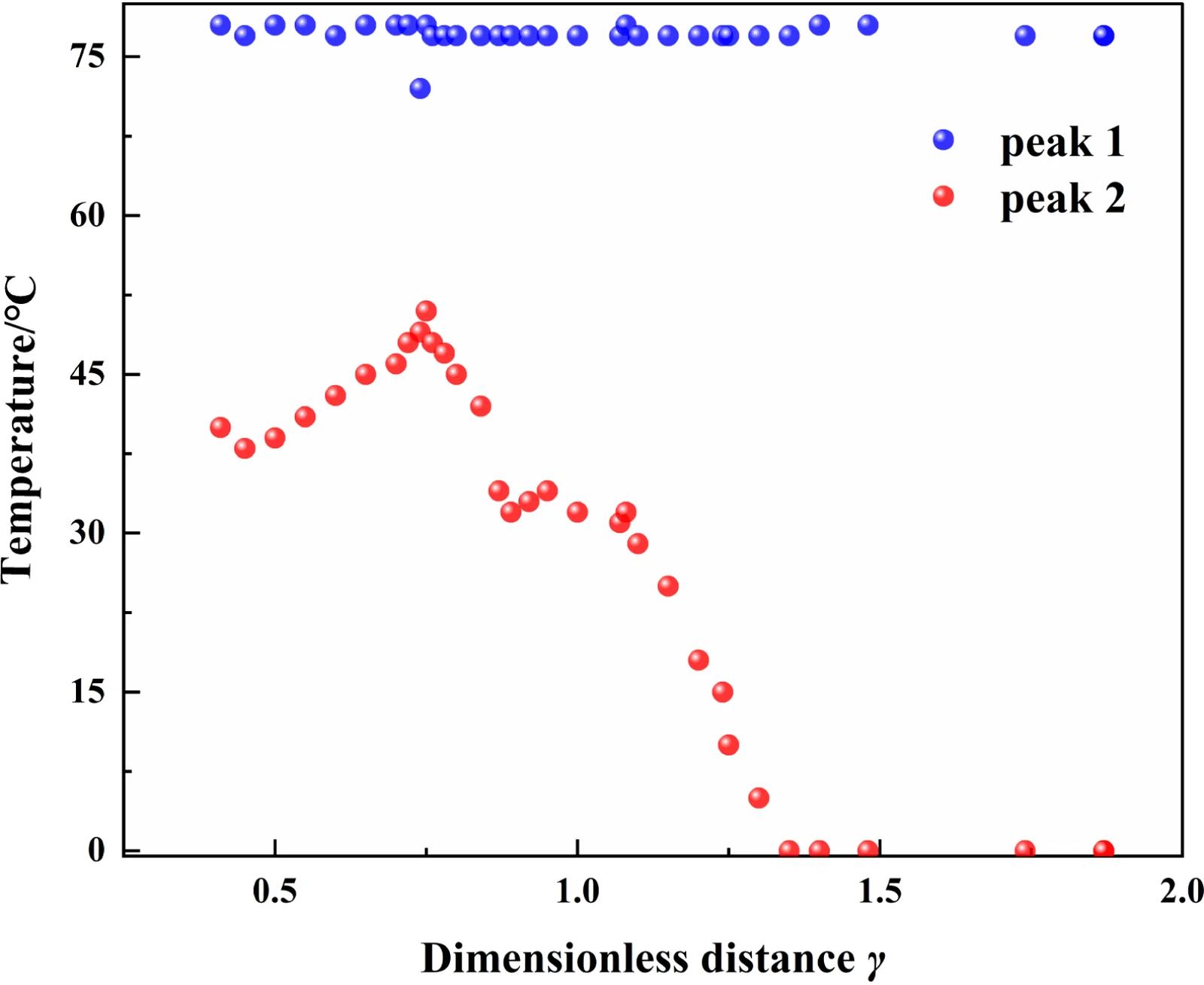
Maximum temperatures of peak 1 and peak 2 as functions of the dimensionless stand-off distance γ.

## 4 Conclusions

In this study, the transient temperature response and its evolution during wall-proximal cavitation bubble collapse were investigated by combining high-speed thin-film thermocouple measurements with hybrid thermal MRT-LBM simulations. Under free-field conditions, the experimentally measured bubble dynamics and temperature evolution were compared with the corresponding numerical predictions. The results validated that the established numerical model can reproduce bubble growth, collapse, and temperature variation, providing a reliable basis for the subsequent analysis of near-wall thermal effects.

The experimental and numerical results show that the transient temperature response during near-wall cavitation bubble collapse can be divided into four stages: the shock-wave-induced temperature stage, the liquid-film compression stage, the cavitation-cooling stage, and the post-cavitation plateau stage. The mechanisms governing the temperature variation in each stage were analyzed individually and supported by the corresponding experimental or numerical evidence. The analysis of the influence of the stand-off distance on the transient temperature response represents a specific application of these mechanisms and further verifies the validity of the proposed interpretation. In particular, peak 2 in the liquid-film compression stage exhibits a pronounced maximum at γ≈0.75.

Two findings of this study are particularly noteworthy. Benefiting from the microsecond-response high-speed thermocouple sensor and the corresponding signal-conditioning and acquisition system jointly developed with our collaborating institutions, we directly measured a shock-wave-induced temperature variation with a rate on the order of 10^6^ K/s and a cavitation-cooling rate on the order of 10^5^ K/s. To the best of our knowledge, this is the first time that the shock-wave-induced temperature-change rate in water and the cavitation-cooling rate have been directly measured using a high-speed thermocouple. Both the 10^6^ K/s shock-wave-induced temperature variation and the 10^5^ K/s cavitation-cooling effect are extreme physical phenomena that may provide an important underlying basis for understanding cavitation erosion, aqueous advanced oxidation, ultrasound-based medical applications, and other cavitation-related processes. Moreover, the observation of these phenomena demonstrates that the proposed direct temperature-measurement scheme based on a microsecond-response high-speed thermocouple is effective and has the potential for broader application.

Further advances in direct temperature-measurement technology will make it increasingly feasible to characterize the transient temperature response during cavitation bubble collapse. Several open questions merit further investigation. Although the present study reveals a remarkable cavitation-cooling effect, the quantitative influences of the governing factors and the methods required to control this cooling precisely remain unclear. In addition, the development of smaller and more mechanically robust sensors may enable the direct measurement of the intrabubble temperature response during the final stage of collapse, providing new experimental evidence for understanding the extreme thermodynamic processes occurring within collapsing cavitation bubbles.

## CRediT authorship contribution statement

**Minglei Shan:** Writing – review & editing, Methodology, Funding acquisition, Conceptualization. **Mengyu Feng:** Writing – original draft, Validation, Investigation. **Ling Kuai:** Writing – original draft, Investigation. **Yu Yang:** Validation, Funding acquisition. **Cheng Yin:** Visualization, Methodology. **Qingbang Han:** Visualization, Validation.

## Declaration of competing interest

The authors declare that they have no known competing financial interests or personal relationships that could have appeared to influence the work reported in this paper.

## Data Availability

The data that support the findings of this study are available from the corresponding author upon reasonable request.
